# Association between hair cortisol concentration and acute stress symptoms in family members of critically ill patients: a cross-sectional study

**DOI:** 10.62675/2965-2774.20240043-en

**Published:** 2024-10-01

**Authors:** Cláudia Severgnini Eugênio, Thiago Wendt Viola, Francisco Sindermann Lumertz, Adriana Valéria Hoffmeister Daltrozo, Maria Eduarda Ramos Saraiva, Júlia Borges Casagrande, Rafael Fernandes Zanin, Márcio Manozzo Boniatti

**Affiliations:** 1 Hospital de Clínicas de Porto Alegre Department of Critical Care Brazil Department of Critical Care, Hospital de Clínicas de Porto Alegre, Brazil.; 2 Universidade Federal do Rio Grande do Sul Porto Alegre RS Brazil Universidade Federal do Rio Grande do Sul - Porto Alegre (RS), Brazil.; 3 Pontifícia Universidade Católica do Rio Grande do Sul Porto Alegre RS Brazil Pontifícia Universidade Católica do Rio Grande do Sul - Porto Alegre (RS), Brazil.; 4 Universidade La Salle Canoas RS Brazil Universidade La Salle - Canoas (RS), Brazil.

**Keywords:** Hydrocortisone, Resilience, psychological, Critical illness, Family, Hair, Prevalence, Intensive care units

## Abstract

**Objective:**

The aim of this study was to investigate whether there is an association between hair cortisol concentrations and acute stress symptoms in family members of critically ill patients.

**Methods:**

A cross-sectional study was conducted in an adult intensive care unit of a tertiary hospital in Porto Alegre, Brazil, from August 2021 to February 2022. Family members of intensive care unit patients admitted for more than 10 days were approached for enrollment. We collected sociodemographic data and assessed resilience, religiosity, and symptoms of acute stress among family members. Samples of family members’ hair were collected shortly after the interview to measure the hair cortisol concentration.

**Results:**

A total of 110 family members were included in this study. Eighty-eight (80.0%) family members presented with symptoms of acute stress. The median hair cortisol concentration was 2.37pg/mg (1.16 - 5.06pg/mg). There was no significant difference in hair cortisol concentration between family members with and without acute stress symptoms (p = 0.419). According to the multivariate analysis, only the fact that the patient was alert at the time of the family member's interview was significantly associated with the prevalence of acute stress symptoms in the family member.

**Conclusion:**

We did not find an association between the hair cortisol concentration of family members in hair segments in the months prior to admission to the intensive care unit and the occurrence of acute stress symptoms.

## INTRODUCTION

Unexpected admission of patients to the intensive care unit (ICU) can be a stressful experience, with serious psychological consequences for family members.^(1)^ The disruption of the daily routine, social, and economic impact of being with the patient during hospitalization, communication problems, the emotional burden associated with decision-making, and an uncertain future for critically ill patients contribute to the psychological distress of family members.^(2-5)^

The physiological and psychological responses to this stressful scenario differ among individuals. The identification of factors contributing to these individual differences in stress responses is of great interest.^(6)^ One factor that potentially influences stress reactivity is chronic alterations in hypothalamic-pituitary-adrenal (HPA) axis activity. The HPA axis regulates the synthesis and release of endocrine hormones, including their end-product, cortisol, which is the main hormone in the stress response system.^(7)^ While a momentary increase in cortisol secretion can play an adaptive role in stressful situations, long-term excessive secretion can have a negative impact on physical and psychological health.^(8)^ Furthermore, attenuation of the HPA axis secondary to previous trauma can interfere with the adaptive response to future traumatic events, thereby increasing the risk of developing stress symptoms.^(9)^ The analysis of hair cortisol concentrations (HCCs) is an advance in this context, as it offers several advantages over traditional methods (blood and saliva) and can be considered a reliable biomarker of long-term HPA axis activity. In addition, HCC represents the endocrine activity of the examinations prior to sample collection, which can be useful information for research that aims to evaluate the activity of the HPA axis before a specific event, such as exposure to trauma.^(10)^

The aim of this study was to investigate whether there is an association between symptoms of acute stress and HCC in family members of critically ill patients.

## METHODS

This cross-sectional study was conducted in an adult ICU at the *Hospital de Clínicas de Porto Alegre*, Rio Grande do Sul, from August 2021 to February 2022. With respect to the visitation policy, family members are allowed visits during three 1-hour periods. Extended visitation is permitted in special circumstances, such as for pediatric patients or those with terminal illnesses. Family members do not participate in multidisciplinary rounds. Family members of patients admitted to the ICU for more than 10 days were eligible for enrollment. We chose to include family members of patients with persistent critical illness because this subgroup potentially faces a greater risk of psychological distress. Longer ICU stays have been identified as a risk factor for family stress, thus justifying our selection criteria. Family members who were younger than 18 years, had hair shorter than 3cm, had cognitive difficulties that made it impossible to understand the questions in the questionnaires, or were using corticosteroids were excluded. The study was approved by the Research Ethics Committee of the *Hospital de Clínicas de Porto Alegre* (CAAE 13552819.2.0000.5327), and informed consent was obtained from all participants.

When the patient completed 10 days of hospitalization in the ICU, the researcher contacted the closest family member for inclusion in the study. We defined close relatives as individuals who spent significant time with the patient and/or identified themselves as caregivers for the critically ill individual. The data collection took place in the ICU interview room, a space that provides privacy and is specifically reserved for discussions between health care professionals and family members. The interviews were conducted after the family members received information about the patient's health status. Sociodemographic variables of family members were collected. Resilience in family members was determined by the Connor–Davidson Resilience Scale.^(11,12)^ This scale is composed of 25 questions, with response options that assume a value ranging from zero to four. The final score ranges from 0 to 100. A score greater than 82 identifies individuals who are resilient.

The Duke University Religion Index (DUREL) was used to assess intrinsic religiosity.^(13,14)^ The scale has 5 questions, with the last three questions comprising the intrinsic religiosity score, which ranges from 3 to 15 points. A score lower than 10 points indicates high intrinsic religiosity.^(15)^

Symptoms of acute stress were measured via the Impact of Events Scale-Revised (IES-R).^(16,17)^ The scale is composed of 22 questions that are evaluated on a 5-point scale ranging from 0 (not at all) to 4 (extremely). The total score ranges from 0 to 88. In this study, a score of 33 was chosen as the cutoff point for acute stress symptoms following the recommendations of other studies.^(17,18)^

Hair samples were collected from family members shortly after the questionnaires were completed. Hair strands approximately 3 - 5mm in diameter and 3cm in length were cut from the position of the posterior vertex of the individuals’ heads via surgical scissors. Assuming average hair growth of approximately 1cm per month, each segment collected represents a period equivalent to 3 months. To assess the period prior to ICU admission, only the most distal 2cm of the hair segment was analyzed. After collection, the scalp end of the sample was identified, and the hair samples were stored at room temperature for up to 12 months. The hair samples were subsequently washed twice with isopropanol and dried for 12 hours. The samples were then minced with clean, fine-tipped surgical scissors into pieces approximately 1 mm in length. The samples were weighed and then incubated with methanol (1.5mL) for 22 hours at 50°C in 15mL tubes. The mixture was vortexed every 8 hours. After incubation, 1.0 mL of the supernatant methanol mixture containing the cortisol extract was removed into a clean microtube and subsequently evaporated at 50°C in a fume hood with the airflow stabilized for 14 hours. The residues were reconstituted in 150 µL of phosphate-buffered saline (pH 8.0) and vortexed for 30 seconds. For the double-blind measurement of cortisol in the extracts, we used a commercially available high-sensitivity salivary cortisol enzyme-linked immunosorbent assay (ELISA) (Salimetrics LLC, State College, PA, USA) according to the manufacturer's instructions. All samples were run in duplicate. The colorimetric reading was taken at 405nm, and the data were interpolated according to the absorbance of the standard curve for each assay, subtracting the absorbance of the zeroed wells. The intra- and inter- assay coefficients of variation for hair cortisol analysis were estimated to be 4.72% and 7.46%, respectively. Cortisol analysis was not possible in six participants because of extraction failure (missing data). The assay readings were converted to picograms (pg) of cortisol per milligram (mg) of dry hair.

Patient data, including age, sex, neurological status at the time of the interview, and the need for mechanical ventilation (MV), vasopressors, or renal replacement therapy (RRT), were also collected.

The sample size was calculated to test the existence of a linear association between cortisol levels and IES-R scores. Considering a power of 90%, with an alpha error of 5% and a moderate effect size (correlation = 0.3), the calculated sample size was 109 individuals.

Categorical variables are presented as numbers and percentages, and continuous variables are presented as the means and standard deviations (SDs) or medians and interquartile ranges (IQRs), as appropriate. The baseline characteristics of family members with and without acute stress symptoms were compared via the chi-square test with standardized residuals adjusted for categorical variables and the t test or Wilcoxon-Mann-Whitney U test for continuous variables, according to normality criteria. The correlation between two continuous variables was assessed via Spearman's correlation test. A logistic regression model was constructed to identify the variables independently associated with acute stress symptoms. In the multivariate analysis, HCC was maintained as a variable of interest. After univariate analysis, variables with p < 0.20 were introduced into the model. A p value < 0.05 was considered statistically significant at the 95% confidence level. Statistical analysis was performed via Statistical Package for the Social Sciences (SPSS), version 20.0 software.

## RESULTS

**Figure 1:**
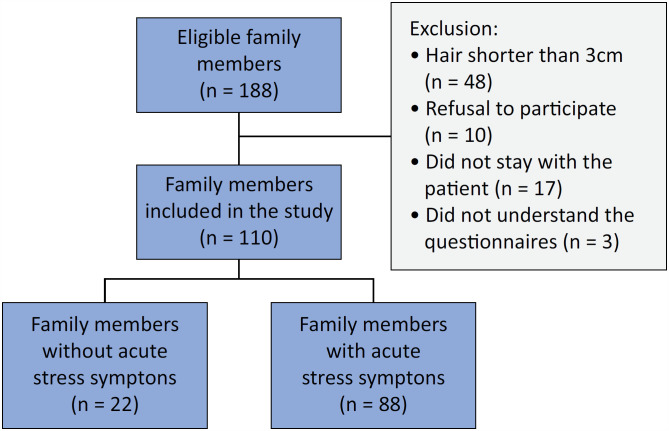
Family member inclusion.

Of the 188 eligible family members, 110 were included in the study. Family members with hair shorter than 3cm (n = 48), those who did not agree to participate in the study (n = 10), those who did not stay with the patient (n = 17), or those who had difficulty understanding the questionnaires (n = 3) were excluded (Figure 1). The characteristics of the family members and patients are presented in table 1. Eighty-eight (80%) family members had symptoms of acute stress. The family members of patients who were unresponsive at the time of the interview had a higher prevalence of acute stress symptoms.

**Table 1:** General characteristics of family members and patients

Variables			Without acute stress symptoms (n = 22)	With acute stress symptoms (n = 88)	p value
Family member					
	Age (years)		53.0 (36.55 - 62.0)	42.5 (30.0 - 57.3)	0.146
	Women		18 (81.8)	80 (90.9)	0.253
	Marital status				0.204
		Single	4 (18.2)	31 (35.2)	
		Married	13 (59.1)	49 (55.7)	
		Divorced	2 (9.1)	4 (4.5)	
		Widowed	3 (13.6)	4 (4.5)	
	Relationship with the patient				0.036
		Children	7 (31.8)	43 (48.9)	
		Spouse	5 (22.7)	17 (19.3)	
		Parents	1 (4.5)	14 (15.9)	
		Siblings	9 (40.9)*	12 (13.6)*	
		Others	–	2 (2.3)	
	Educational level				0.436
		Elementary school	11 (50.0)	31 (35.2)	
		Senior high school	7 (31.8)	38 (43.2)	
		University	4 (18.2)	19 (21.6)	
	Race				0.514
		White	13 (59.1)	62 (70.5)	
		Brown	4 (18.2)	14 (15.9)	
		Black	5 (22.7)	12 (13.6)	
	Working		13 (59.1)	54 (61.4)	0.845
	History of depression, anxiety or PTSD		–	8 (9.1)	0.354
	Psychoactive medication use		–	9 (10.2)	0.200
	High intrinsic religiosity		19 (86.4)	78 (88.6)	0.721
	Resilient		5 (22.7)	24 (27.3)	0.791
Patients					
	Age (years)		59.5 (45.00 - 67.3)	59.0 (46.3 - 69.0)	0.811
	Women		9 (40.9)	37 (42.0)	0.923
	Previous ICU admissions		11 (50.0)	39 (44.3)	0.632
	Mechanical ventilation		20 (90.9)	80 (90.9)	1.000
	Vasopressor		19 (86.4)	81 (92.0)	0.415
	Renal replacement therapy		10 (45.5)	43 (48.9)	0.775
	Length of ICU stay (days)		17.5 (10.8 - 41.5)	19.0 (13.0 - 29.8)	0.791
	Neurological assessment				0.029
		Alert	14 (63.6)*	26 (29.5)*	
		Responds to voice	2 (9.1)	11 (12.5)	
		Responds to pain	1 (4.5)	9 (10.2)	
		Unresponsive	5 (22.7)*	42 (47.7)*	

PTSD - posttraumatic stress disorder; ICU - intensive care unit.

* Standardized residual > 1.96. The results are expressed as the median (interquartile range) or n (%).

Only 29 family members (26.4%) were considered resilient. The characteristics of family members, categorized by the presence of resilience, are described in table 2. Ninety-seven (88.2%) family members had high intrinsic religiosity. There was no association between intrinsic religiosity and stress symptoms (p = 0.721) or between resilience and stress symptoms (p = 0.791). A significant correlation was found between high intrinsic religiosity and resilience (rho -0.375; p < 0.001).

**Table 2:** Characteristics of family members according to resilience

		Not resilient (n = 81)	Resilient (n = 29)	p value
Age (years)		43.0 (30.0 - 56.0)	50.0 (35.0 - 60.0)	0.209
Women		71 (87.7)	27 (93.1)	0.512
Marital status				0.642
	Single	25 (30.9)	10 (34.5)	
	Married	48 (59.3)	14 (48.3)	
	Divorced	4 (4.9)	2 (6.9)	
	Widowed	4 (4.9)	3 (10.3)	
Relationship with the patient				0.861
	Children	37 (45.7)	13 (44.8)	
	Spouse	15 (18.5)	7 (24.1)	
	Parents	12 (14.8)	3 (10.3)	
	Siblings	16 (19.8)	5 (17.2)	
	Others	1 (1.2)	1 (3.4)	
Educational level				0.085
	Elementary school	30 (37.0)	12 (41.4)	
	Senior high school	30 (37.0)	15 (51.7)	
	University	21 (25.9)	2 (6.9)	
Race				0.558
	White	53 (65.4)	22 (75.9)	
	Brown	14 (17.3)	4 (13.8)	
	Black	14 (17.3)	3 (10.3)	
Working		53 (65.4)	14 (48.3)	0.124

The results are expressed as the median (interquartile range) or n (%).

**Figure 2:**
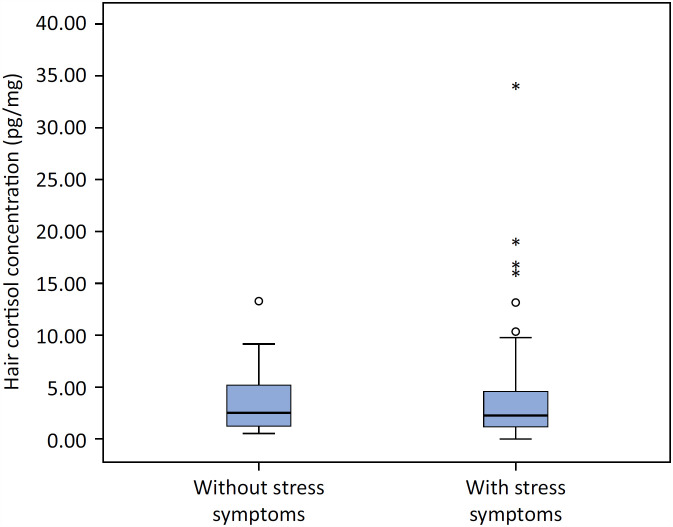
Hair cortisol concentration among family members with and without stress symptoms.

The median HCC was 2.37pg/mg (1.16 - 5.06pg/mg). For 6 participants, it was not possible to perform cortisol analysis due to extraction failure. There was no difference in HCC between family members with and without symptoms of acute stress (p = 0.419) (Figure 2). There was also no difference in HCC in relation to sex (p = 0.483) or age (p = 0.385) of the family members. The HCC differed according to ethnicity (p = 0.023). The black family members had lower HCC (1.28pg/mg; 0.43 - 2.49pg/mg) compared to white or brown family members (2.54pg/mg; 1.24 - 5.31pg/mg).

According to the multivariate analysis, only the fact that the patient was alert at the time of the family member's interview was significantly associated with the prevalence of acute stress symptoms in the family member (Table 3).

**Table 3:** Multivariate analysis of the prevalence of acute stress symptoms

Variables	OR	95%CI	p value
Family member's age	0.980	0.946 - 1.015	0.258
Relationship with the patient - Sibling	0.422	0.122 - 1.457	0.172
Neurological assessment of the patient - Alert	0.281	0.096 - 0.826	0.021
Hair cortisol concentration	1.012	0.899 - 1.140	0.842

OR - odds ratio; 95%CI - 95% confidence interval. Variables with p values < 0.20 in the univariate analysis were entered simultaneously into the regression model. The model retained hair cortisol concentration as a variable of interest.

## DISCUSSION

We found a high prevalence of acute stress symptoms among the family members of patients with persistent critical illness. The HCC of family members in hair segments referred to the months prior to the patient's admission to the ICU was not useful for predicting the occurrence of these symptoms.

Previous studies have suggested that stress may be related to complex dysregulation of the HPA axis. Regarding the basal secretion of cortisol, a meta-analysis revealed hypocortisolism in patients with posttraumatic stress disorder (PTSD) and an increase in cortisol secretion in patients with acute stress.^(19)^ The following two-phase process of endocrine changes in response to traumatic stress has been suggested. Initially, exposure to trauma leads to an increase in cortisol secretion. However, in the second phase, there is a dose-dependent attenuation of cortisol over time, which in turn may predispose individuals to the development of stress upon exposure to a new traumatic event.^(20-22)^ The proposed mechanism by which decreased cortisol secretion may increase the risk of stress after trauma involves the effects of glucocorticoids on stress-related memory processes. After trauma, attenuated cortisol levels can induce a neuroendocrine environment of inadequate consolidation of traumatic memories.^(23,24)^ Thus, prolonged hyposecretion of the HPA axis secondary to previous trauma could interfere with the generation of stress during future traumatic events.^(9)^ Consistent with this hypothesis, Steudte-Schmiedgen et al. reported that lower baseline levels of hair cortisol were associated with an increase in stress symptomatology in soldiers who experienced a new traumatic event.^(20)^ In our study, however, the HCC of family members in hair segments referred to the months prior to the patient's admission to the ICU was not useful for predicting the occurrence of stress symptoms. The lack of a significant association between psychological stress and hair cortisol is consistent with the findings of previous studies.^(25,26)^

Symptoms of anxiety, depression, and acute stress are common among the relatives of critically ill patients.^(5,27,28)^ In addition to the uncertainties experienced in relation to patients’ prognosis, family members must make life-or-death therapeutic decisions on behalf of their disabled loved ones at a time when they themselves are distressed. The prevalence of PTSD symptoms among relatives of critically ill patients is estimated to range from 33 - 56%.^(29-32)^ The incidence of acute stress can be even greater when the evaluation is carried out with family members during their ICU stay. Pillai et al. reported a prevalence of acute stress of 72%.^(33)^ More recently, Zante et al. reported a prevalence of 90% in the relatives of patients admitted to the ICU during the coronavirus disease 2019 (COVID-19) pandemic.^(34)^ Our findings confirmed the high prevalence of acute stress symptoms in the relatives of critically ill patients. This high prevalence may also be related to the fact that we included patients with persistent critical illness, as a longer ICU stay was identified as a risk factor for family stress.^(35)^

Considering the high prevalence of acute stress and/or PTSD in the relatives of critically ill patients, it is very important to identify those at greater risk of developing symptoms. Wendlandt et al. reported that the level of stress symptoms at study inclusion (during the ICU) was a predictor of the level of stress symptoms at subsequent assessments (3 and 6 months).^(36)^ This finding of a fixed trajectory of stress symptoms suggests that family-specific factors, rather than patient-specific factors, could play an important role in this trajectory. However, our findings did not support this hypothesis. The only factor associated with acute stress symptoms in family members was the patient's neurological condition at the time of the interview. Relatives from unresponsive patients presented a greater occurrence of acute stress symptoms. Barth et al. verified that patients being in a state of coma and being unable to speak were important stressors for their relatives.^(37)^

Our study has several limitations. First, we included only family members of patients who stayed in the ICU for > 10 days, making it impossible to extrapolate these findings to all family members of critically ill patients. In addition, we did not investigate stressful situations experienced by family members prior to the patient's admission to the ICU. The HCC referred to 2 months prior to hospitalization may not necessarily be the baseline HCC of the family member, since other types of stressful situations, in addition to the health condition of the future patient, could have occurred during this period. Furthermore, a history of anxiety, depression or posttraumatic stress was self-reported, which may have led to an underestimation of the prevalence of a history of psychiatric illness in this population. Finally, we did not collect information about hair treatment or washing frequency. However, the associations between these variables and HCC incidence appear to be weak. ^(19)^

## CONCLUSION

We did not find an association between the hair cortisol concentration of family members in hair segments in the months prior to the patient's admission to the intensive care unit and the occurrence of acute stress symptoms. Psychological distress and hypothalamic-pituitary-adrenal axis reactivity among family members of critically ill patients deserves further study. In addition, other studies must be conducted to investigate the characteristics that contribute to a greater risk of stress among the relatives of critically ill patients.

## DATA AVAILABILITY

The datasets used and/or analyzed during the current study are available from the corresponding author upon reasonable request.
