# Supplementary material for: Association between hair cortisol concentration and acute stress symptoms in family members of critically ill patients: a cross-sectional study
Source: Crit Care Sci. 2024 Oct 1;36:e20240043en. doi: 10.62675/2965-2774.20240043-en (PMC11463979; doi:10.62675/2965-2774.20240043-en)
Supplement: Translation

# Translation: Associação entre concentração de cortisol capilar e sintomas de estresse agudo em familiares de pacientes graves: um estudo transversal

**DOI:** 10.62675/2965-2774.20240043-pt

**Authors:** Cláudia Severgnini Eugênio, Thiago Wendt Viola, Francisco Sindermann Lumertz, Adriana Valéria Hoffmeister Daltrozo, Maria Eduarda Ramos Saraiva, Júlia Borges Casagrande, Rafael Fernandes Zanin, Márcio Manozzo Boniatti

**Affiliations:** 1 Brasil Departamento de Cuidados Críticos, Hospital de Clínicas de Porto Alegre, Brasil.; 2 Porto Alegre RS Brasil Universidade Federal do Rio Grande do Sul - Porto Alegre (RS), Brasil.; 3 Porto Alegre RS Brasil Pontifícia Universidade Católica do Rio Grande do Sul - Porto Alegre (RS), Brasil.; 4 Canoas RS Brasil Universidade La Salle - Canoas (RS), Brasil.

**Keywords:** Hidrocortisona, Resiliência psicológica, Estado terminal, Família, Cabelo, Prevalência, Unidades de terapia intensiva

## Abstract

**Objetivo:**

Este estudo pretendeu investigar se existe uma associação entre concentrações de cortisol capilar e sintomas de estresse agudo em familiares de pacientes graves.

**Métodos:**

Trata-se de estudo transversal realizado em uma unidade de terapia intensiva de adultos de um hospital terciário em Porto Alegre (RS), de agosto de 2021 a fevereiro de 2022. Os familiares de pacientes internados em uma unidade de terapia intensiva por mais de 10 dias foram abordados para inscrição. Coletamos dados sociodemográficos e avaliamos a resiliência, a religiosidade e os sintomas de estresse agudo entre os familiares. Coletaram-se amostras de cabelo dos familiares logo após a entrevista para medir a concentração de cortisol capilar.

**Resultados:**

O estudo incluiu 110 familiares. Foram identificados sintomas de estresse agudo em 88 (80,0%) familiares. A mediana da concentração de cortisol capilar foi de 2,37pg/mg (1,16 - 5,06pg/mg). Não houve diferença significativa na concentração de cortisol capilar entre os familiares com e sem sintomas de estresse agudo (p = 0,419). De acordo com a análise multivariada, apenas o fato de o paciente estar alerta no momento da entrevista do familiar foi significativamente associado à prevalência de sintomas de estresse agudo no familiar.

**Conclusão:**

Não encontramos uma associação entre a concentração de cortisol capilar dos familiares em segmentos de cabelo nos meses anteriores à admissão na unidade de terapia intensiva e a ocorrência de sintomas de estresse agudo.

## INTRODUÇÃO

A admissão inesperada de pacientes na unidade de terapia intensiva (UTI) pode ser uma experiência estressante, com sérias consequências psicológicas para os familiares.^(1)^ A interrupção da rotina diária, o impacto social e econômico de estar com o paciente durante a hospitalização, os problemas de comunicação, a carga emocional associada à tomada de decisões e um futuro incerto para pacientes graves contribuem para o sofrimento psicológico dos familiares.^(2-5)^

As respostas fisiológicas e psicológicas a esse cenário estressante diferem entre os indivíduos. É de grande interesse a identificação dos fatores que contribuem para essas diferenças individuais nas respostas ao estresse.^(6)^ Um fator que potencialmente influencia a reatividade ao estresse são as alterações crônicas na atividade do eixo hipotálamo-pituitária-adrenal (HPA). O eixo HPA regula a síntese e a liberação de hormônios endócrinos, inclusive seu produto final, o cortisol, que é o principal hormônio no sistema de resposta ao estresse.^(7)^ Embora um aumento momentâneo na secreção de cortisol possa desempenhar papel adaptativo em situações estressantes, a secreção excessiva a longo prazo pode ter efeito negativo na saúde física e psicológica.^(8)^ Além disso, a atenuação do eixo HPA secundária a traumas anteriores pode interferir na resposta adaptativa a eventos traumáticos futuros, aumentando o risco de desenvolver sintomas de estresse.^(9)^ A análise do nível de cortisol capilar (CHC) é um avanço nesse contexto, pois oferece várias vantagens em relação aos métodos tradicionais (sangue e saliva) e pode ser considerado um biomarcador confiável da atividade do eixo HPA em longo prazo. Além disso, o CHC representa a atividade endócrina dos exames antes da coleta da amostra, o que pode ser uma informação útil para pesquisas que visam avaliar a atividade do eixo HPA antes de um evento específico, como a exposição a um trauma.^(10)^

Este estudo pretendeu investigar se existe uma associação entre sintomas de estresse agudo e CHC em familiares de pacientes críticos.

## MÉTODOS

Trata-se de estudo transversal realizado em uma UTI adulta do Hospital de Clínicas de Porto Alegre (RS), de agosto de 2021 a fevereiro de 2022. Com relação à política de visitação, os familiares tinham permissão para visitar durante três períodos de 1 hora cada. A visitação estendida é permitida em circunstâncias especiais, como para pacientes pediátricos ou com doenças terminais. Os familiares não participam de rodadas multidisciplinares. Os familiares de pacientes internados na UTI por mais de 10 dias eram considerados aptos a se inscreverem. Optamos por incluir os familiares de pacientes com doença crítica persistente porque esse subgrupo pode apresentar um risco maior de sofrimento psicológico. Internações mais longas na UTI foram identificadas como um fator de risco para o estresse familiar, justificando nossos critérios de seleção. Foram excluídos familiares menores de 18 anos, com cabelos com menos de 3cm, com dificuldades cognitivas que impossibilitassem a compreensão das perguntas dos questionários ou que estivessem usando corticosteroides. O estudo foi aprovado pelo Comitê de Ética em Pesquisa do Hospital de Clínicas de Porto Alegre (CAAE 13552819.2.0000.5327), e obteve-se o termo de Consentimento Livre e Esclarecido de todos os participantes.

Ao completar 10 dias de hospitalização na UTI, o pesquisador contatava o familiar mais próximo para inclusão no estudo. Definimos parentes próximos ao paciente os indivíduos que passassem um tempo significativo com o paciente e/ou se identificassem como cuidadores do indivíduo grave. A coleta de dados foi realizada na sala de entrevistas da UTI, um espaço que oferece privacidade e é reservado especificamente para discussões entre profissionais de saúde e familiares. As entrevistas eram realizadas após os familiares receberem informações do estado de saúde do paciente. Foram coletadas variáveis sociodemográficas dos familiares. A resiliência dos familiares foi determinada pela escala de resiliência Connor-Davidson.^(11,12)^ Essa escala é composta de 25 perguntas, com opções de resposta que assumem um valor que varia de zero a quatro. A pontuação final varia de zero a cem. Escore superior a 82 identifica indivíduos resilientes.

O *Duke University Religion Index* (DUREL) foi usado para avaliar a religiosidade intrínseca.^(13,14)^ A escala tem 5 perguntas, sendo que as 3 últimas compõem o escore de religiosidade intrínseca, que varia de 3 a 15 pontos. Pontuação inferior a dez pontos indica alta religiosidade intrínseca.^(15)^

Os sintomas de estresse agudo foram medidos por meio da *Impact of Events Scale - Revised* (IES-R).^(16,17)^ A escala é composta de 22 perguntas que são avaliadas em uma escala de 5 pontos que varia de zero (nem um pouco) a 4 (extremamente). A pontuação total varia de zero a 88. Neste estudo, escolheu-se como ponto de corte para sintomas de estresse agudo pontuação de 33, seguindo as recomendações de outros estudos.^(17,18)^

As amostras de cabelo foram coletadas dos familiares logo após o preenchimento dos questionários. As mechas de cabelo com aproximadamente 3 a 5mm de diâmetro e 3cm de comprimento foram cortadas da posição do vértice posterior da cabeça dos indivíduos com uma tesoura cirúrgica. Considerando que o crescimento médio do cabelo é de aproximadamente 1cm por mês, cada segmento coletado representa um período equivalente a 3 meses. Para avaliar o período anterior à admissão na UTI, apenas os 2cm mais distais do segmento de cabelo foram analisados. Após a coleta, a extremidade do couro cabeludo da amostra foi identificada, e as amostras de cabelo foram armazenadas em temperatura ambiente por até 12 meses. Em seguida, as amostras de cabelo foram lavadas duas vezes com isopropanol e secas por 12 horas. Em seguida, as amostras foram picadas com uma tesoura cirúrgica limpa e de ponta fina em pedaços de aproximadamente 1mm de comprimento. As amostras foram pesadas e incubadas com metanol (1,5mL) por 22 horas a 50°C em tubos de 15mL. A mistura foi agitada em vórtex a cada 8 horas. Após a incubação, retirou-se 1mL da mistura de metanol sobrenadante contendo o extrato de cortisol em um microtubo limpo e, em seguida, evaporou-se a 50°C em uma capela de exaustão com o fluxo de ar estabilizado por 14 horas. Os resíduos foram reconstituídos em 150µL de solução salina tamponada com fosfato (pH 8,0) e agitados em vórtex por 30 segundos. Na medição duplo-cega do cortisol nos extratos, usamos um ensaio de imunoabsorção enzimática (ELISA) de cortisol salivar de alta sensibilidade disponível comercialmente (Salimetrics LLC, State College, PA, Estados Unidos), seguindo as instruções do fabricante. Todas as amostras foram analisadas em duplicata. A leitura colorimétrica foi feita a 405nm, e os dados foram interpolados conforme a absorbância da curva-padrão para cada ensaio, subtraindo a absorbância dos poços zerados. Os coeficientes de variação intra e interensaio na análise do cortisol capilar foram estimados em 4,72 e 7,46%, respectivamente. A análise de cortisol não foi possível em seis participantes devido a falhas na extração (dados ausentes). As leituras do ensaio foram convertidas em picogramas (pg) de cortisol por miligrama (mg) de cabelo seco.

Coletamos também dados dos pacientes, inclusive idade, sexo, estado neurológico no momento da entrevista e necessidade de ventilação mecânica (VM), vasopressores ou terapia de substituição renal (TSR).

O tamanho da amostra foi calculado para testar a existência de uma associação linear entre os níveis de cortisol e os escores do IES-R. Considerando poder de 90%, com erro alfa de 5% e tamanho de efeito moderado (correlação = 0,3), o tamanho da amostra calculado foi de 109 indivíduos.

As variáveis categóricas são apresentadas como número e percentual e as variáveis contínuas, como média e desvio-padrão (DP) ou mediana e intervalo interquartil (IQ25 - 75%), conforme o caso. As características basais dos familiares com e sem sintomas de estresse agudo foram comparadas por meio do teste qui-quadrado com resíduos padronizados ajustados para variáveis categóricas e o teste *t* ou o teste U de Wilcoxon-Mann-Whitney para variáveis contínuas, obedecendo aos critérios de normalidade. A correlação entre duas variáveis contínuas foi avaliada pelo teste de correlação de Spearman. Elaborou-se um modelo de regressão logística para identificar as variáveis independentemente associadas aos sintomas de estresse agudo. Na análise multivariada, o CHC foi mantido como variável de interesse. Após a análise univariada, as variáveis com p < 0,20 foram introduzidas no modelo. Valor de p < 0,05 foi considerado estatisticamente significativo em um nível de confiança de 95%. A análise estatística foi realizada com o *software Statistical Package for the Social Sciences* (SPSS), versão 20.0.

## RESULTADOS

Dos 188 familiares aptos a participar, 110 foram incluídos no estudo. Foram excluídos familiares com cabelo menor do que 3cm (n = 48), os que não concordaram em participar do estudo (n = 10), os que não ficavam com o paciente (n = 17) ou os que tiveram dificuldade para entender os questionários (n = 3) (Figura 1). As características dos familiares e dos pacientes são apresentadas na tabela 1. Em 80% (n = 88) dos casos, os familiares apresentavam sintomas de estresse agudo. Os familiares de pacientes que não estavam respondendo no momento da entrevista apresentaram maior prevalência de sintomas de estresse agudo.

**Figura 1:** Inclusão de familiares.

**Tabela 1:** Características gerais dos familiares e dos pacientes

Variáveis			Sem sintomas de estresse agudo (n = 22)	Com sintomas de estresse agudo (n = 88)	Valor de p
Familiares					
	Idade (anos)		53,0 (36,55 - 62,0)	42,5 (30,0 - 57,3)	0,146
	Mulheres		18 (81,8)	80 (90,9)	0,253
	Estado civil				0,204
		Solteiro	4 (18,2)	31 (35,2)	
		Casado	13 (59,1)	49 (55,7)	
		Divorciado	2 (9,1)	4 (4,5)	
		Viúvo	3 (13,6)	4 (4,5)	
	Vínculo com o paciente				0,036
		Filhos	7 (31,8)	43 (48,9)	
		Cônjuge	5 (22,7)	17 (19,3)	
		Pais	1 (4,5)	14 (15,9)	
		Irmãos	9 (40,9)*	12 (13,6)*	
		Outros	-	2 (2,3)	
	Escolaridade				0,436
		Ensino Fundamental	11 (50,0)	31 (35,2)	
		Ensino Médio	7 (31,8)	38 (43,2)	
		Ensino Superior	4 (18,2)	19 (21,6)	
	Cor da pele				0,514
		Branca	13 (59,1)	62 (70,5)	
		Parda	4 (18,2)	14 (15,9)	
		Negra	5 (22,7)	12 (13,6)	
	Em atividade laboral		13 (59,1)	54 (61,4)	0,845
	Histórico de depressão, ansiedade ou TEPT		-	8 (9,1)	0,354
	Uso de medicamentos psicoativos		-	9 (10,2)	0,200
	Alta religiosidade intrínseca		19 (86,4)	78 (88,6)	0,721
	Resilientes		5 (22,7)	24 (27,3)	0,791
Pacientes					
	Idade (anos)		59,5 (45,00 - 67,3)	59,0 (46,3 - 69,0)	0,811
	Mulheres		9 (40,9)	37 (42,0)	0,923
	Internações prévias em UTI		11 (50,0)	39 (44,3)	0,632
	Ventilação mecânica		20 (90,9)	80 (90,9)	1,000
	Vasopressores		19 (86,4)	81 (92,0)	0,415
	Terapia de substituição renal		10 (45,5)	43 (48,9)	0,775
	Tempo de internação na UTI (dias)		17,5 (10,8 - 41,5)	19,0 (13,0 - 29,8)	0,791
	Avaliação neurológica				0,029
		Alerta	14 (63,6)*	26 (29,5)*	
		Responde à vocalização	2 (9,1)	11 (12,5)	
		Responde à dor	1 (4,5)	9 (10,2)	
		Não responsivo	5 (22,7)*	42 (47,7)*	

TEPT - transtorno de estresse pós-traumático; UTI - unidade de terapia intensiva.

* Resíduo padronizado > 1,96. Resultados expressos como mediana (intervalo interquartil) ou n (%).

Apenas 29 familiares (26,4%) foram considerados resilientes. As características dos familiares, categorizadas pela presença de resiliência, estão descritas na tabela 2. A religiosidade intrínseca de 97 (88,2%) familiares era alta. Não houve associação entre religiosidade intrínseca e sintomas de estresse (p = 0,721) ou entre resiliência e sintomas de estresse (p = 0,791). Encontrou-se correlação significativa entre alta religiosidade intrínseca e resiliência (rho -0,375; p < 0,001).

**Tabela 2:** Características dos familiares quanto à resiliência

		Não resilientes (n = 81)	Resilientes (n = 29)	Valor de p
Idade (anos)		43,0 (30,0 - 56,0)	50,0 (35,0 - 60,0)	0,209
Mulheres		71 (87,7)	27 (93,1)	0,512
Estado civil				0,642
	Solteiro	25 (30,9)	10 (34,5)	
	Casado	48 (59,3)	14 (48,3)	
	Divorciado	4 (4,9)	2 (6,9)	
	Viúvo	4 (4,9)	3 (10,3)	
Vínculo com o paciente				0,861
	Filhos	37 (45,7)	13 (44,8)	
	Cônjuge	15 (18,5)	7 (24,1)	
	Pais	12 (14,8)	3 (10,3)	
	Irmãos	16 (19,8)	5 (17,2)	
	Outros	1 (1,2)	1 (3,4)	
Escolaridade				0,085
	Ensino Fundamental	30 (37,0)	12 (41,4)	
	Ensino Médio	30 (37,0)	15 (51,7)	
	Ensino Superior	21 (25,9)	2 (6,9)	
Cor da pele				0,558
	Branca	53 (65,4)	22 (75,9)	
	Parda	14 (17,3)	4 (13,8)	
	Negra	14 (17,3)	3 (10,3)	
Em atividade laboral		53 (65,4)	14 (48,3)	0,124

Resultados são expressos como mediana (intervalo interquartil) ou n (%).

A mediana do CHC foi de 2,37pg/mg (1,16 - 5,06pg/mg). Não foi possível realizar a análise do CHC de seis participantes, devido a falhas na extração. Não houve diferença no CHC entre os familiares com e sem sintomas de estresse agudo (p = 0,419) (Figura 2). Também não houve diferença no CHC em relação ao sexo (p = 0,483) ou à idade (p = 0,385) dos familiares. As diferenças no CHC foram em relação à cor da pele (p = 0,023). Os familiares negros apresentaram menor CHC (1,28pg/mg; 0,43 - 2,49pg/mg) em comparação com os familiares brancos ou pardos (2,54pg/mg; 1,24 - 5,31pg/mg).

**Figura 2:** Nível de cortisol capilar em familiares com e sem sintomas de estresse.

De acordo com a análise multivariada, apenas o fato de o paciente estar alerta no momento da entrevista do familiar foi significativamente associado à prevalência de sintomas de estresse agudo no familiar (Tabela 3).

**Tabela 3:** Análise multivariada da prevalência de sintomas de estresse agudo

Variáveis	RC	IC95%	Valor de p
Idade do familiar	0,980	0,946 - 1,015	0,258
Vínculo com o paciente - irmãos	0,422	0,122 - 1,457	0,172
Avaliação neurológica do paciente - alerta	0,281	0,096 - 0,826	0,021
Nível de cortisol capilar	1,012	0,899 - 1,140	0,842

RC - razão de chances; IC95% - intervalo de confiança de 95%. As variáveis com valores de p < 0,20 na análise univariada foram inseridas simultaneamente no modelo de regressão. O modelo manteve o nível de cortisol capilar como uma variável de interesse.

## DISCUSSÃO

Encontramos alta prevalência de sintomas de estresse agudo entre os familiares de pacientes com doença crítica persistente. O CHC dos familiares em segmentos capilares referentes aos meses anteriores à admissão do paciente na UTI não foi útil para prever a ocorrência desses sintomas.

Estudos anteriores sugeriram que o estresse pode estar relacionado à desregulação complexa do eixo HPA. Em relação à secreção basal de cortisol, uma metanálise revelou hipocortisolismo em pacientes com transtorno de estresse pós-traumático (TEPT) e um aumento na secreção de cortisol em pacientes com estresse agudo.^(19)^ Sugeriu-se o seguinte processo de duas fases de alterações endócrinas em resposta ao estresse traumático. Inicialmente, a exposição ao trauma leva a um aumento na secreção de cortisol. No entanto, na segunda fase, há uma atenuação dose-dependente do cortisol ao longo do tempo, o que, por sua vez, pode predispor os indivíduos ao desenvolvimento de estresse após a exposição a um novo evento traumático.^(20-22)^ O mecanismo proposto pelo qual a diminuição da secreção de cortisol pode aumentar o risco de estresse pós-traumático envolve os efeitos dos glicocorticoides nos processos de memória relacionados ao estresse. Após o trauma, os níveis atenuados de cortisol podem induzir um ambiente neuroendócrino de consolidação inadequada de memórias traumáticas.^(23,24)^ Assim, a hipossecreção prolongada do eixo HPA secundária a um trauma anterior poderia interferir na geração de estresse durante eventos traumáticos futuros.^(9)^ Em consonância com essa hipótese, Steudte-Schmiedgen et al. relataram que CHCs basais mais baixos estavam associados a um aumento na sintomatologia de estresse em soldados que passaram por um novo evento traumático.^(20)^ Em nosso estudo, entretanto, o CHC de familiares em segmentos de cabelo referentes aos meses anteriores à admissão do paciente na UTI não foi útil para prever a ocorrência de sintomas de estresse. A falta de uma associação significativa entre estresse psicológico e CHC é consistente com os achados de estudos anteriores.^(25,26)^

Sintomas de ansiedade, depressão e estresse agudo são comuns entre os familiares de pacientes graves.^(5,27,28)^ Além das incertezas experimentadas em relação ao prognóstico dos pacientes, os familiares precisam tomar decisões terapêuticas de vida ou morte em nome de seus entes queridos incapacitados em um momento em que eles próprios estão angustiados. Estima-se que a prevalência de sintomas de TEPT entre os familiares de pacientes graves varie de 33 a 56%.^(29-32)^ A incidência de estresse agudo pode ser ainda maior quando a avaliação é realizada com os familiares durante a internação na UTI. Pillai et al. relataram prevalência de estresse agudo de 72%.^(33)^ Recentemente, Zante et al. relataram prevalência de 90% nos familiares de pacientes internados na UTI durante a pandemia da doença pelo coronavírus 2019 (COVID-19).^(34)^ Nossos achados confirmaram a alta prevalência de sintomas de estresse agudo em familiares de pacientes graves. Essa alta prevalência também pode estar relacionada ao fato de termos incluído pacientes com doença crítica persistente, pois uma internação mais longa na UTI foi identificada como fator de risco para o estresse familiar.^(35)^

Considerando a alta prevalência de estresse agudo e/ou TEPT nos familiares de pacientes críticos, é muito importante identificar aqueles com maior risco de desenvolver sintomas. Wendlandt et al. relataram que o nível de sintomas de estresse na inclusão do estudo (durante a UTI) foi preditor do nível de sintomas de estresse nas avaliações subsequentes (3 e 6 meses).^(36)^ Esse achado de uma trajetória fixa de sintomas de estresse sugere que fatores familiares específicos, em vez de fatores específicos do paciente, poderiam desempenhar papel importante nessa trajetória. No entanto, nossos achados não corroboraram essa hipótese. O único fator associado a sintomas de estresse agudo em familiares foi a condição neurológica do paciente no momento da entrevista. Os familiares de pacientes que não responderam apresentaram maior incidência de sintomas de estresse agudo. Barth et al. verificaram que o fato de os pacientes estarem em estado de coma e impossibilitados de falar eram importantes fatores de estresse para seus familiares.^(37)^

Nosso estudo tem várias limitações. Primeiro, incluímos apenas familiares de pacientes que permaneceram na UTI por mais de 10 dias, o que impossibilita a extrapolação desses achados para todos os familiares de pacientes graves. Além disso, não investigamos situações estressantes vivenciadas pelos familiares antes da admissão do paciente na UTI. O CHC referente a 2 meses antes da hospitalização pode não ser necessariamente o CHC basal do familiar, já que outros tipos de situações estressantes, além da condição de saúde do futuro paciente, podem ter ocorrido durante esse período. Além disso, um histórico de ansiedade, depressão ou estresse pós-traumático foi relatado pelo próprio paciente, o que pode ter levado a uma subestimação da prevalência de um histórico de doença psiquiátrica nessa população. Por fim, não coletamos informações quanto ao tratamento dos cabelos ou à frequência de lavagem. Entretanto, as associações entre essas variáveis e a incidência de CHC parecem ser fracas.^(19)^

## CONCLUSÃO

Não encontramos uma associação entre o nível de cortisol capilar dos familiares em segmentos de cabelo nos meses anteriores à admissão do paciente na unidade de terapia intensiva e a ocorrência de sintomas de estresse agudo. A angústia psicológica e a reatividade do eixo hipotálamo-hipófise-adrenal entre os familiares de pacientes graves merecem um estudo mais aprofundado. Além disso, outros estudos devem ser realizados para investigar as características que contribuem para um maior risco de estresse entre os familiares de pacientes graves.

## DISPONIBILIDADE DE DADOS

Os conjuntos de dados usados e/ou analisados durante o presente estudo estão disponíveis com o autor correspondente mediante solicitação razoável.
